# CES1 Increases Hepatic Triacylglycerol Synthesis Through Activation of PPARγ, LXR and SREBP1c

**DOI:** 10.3390/cells14191548

**Published:** 2025-10-03

**Authors:** Rajakumar Selvaraj, Jihong Lian, Russell Watts, Randal Nelson, Michael F. Saikali, Carolyn L. Cummins, Richard Lehner

**Affiliations:** 1 Group on Molecular and Cell Biology of Lipids, Faculty of Medicine & Dentistry, University of Alberta, Edmonton, AB T6G 2S2, Canadajlian1@ualberta.ca (J.L.); rwatts@ualberta.ca (R.W.);; 2 Department of Pediatrics, University of Alberta, Edmonton, AB T6G 2S2, Canada; 3 Department of Pharmaceutical Sciences, Leslie Dan Faculty of Pharmacy, University of Toronto, Toronto, ON M5S 3M2, Canadacarolyn.cummins@utoronto.ca (C.L.C.); 4 Department of Cell Biology, University of Alberta, Toronto, AB T6G 2S2, Canada

**Keywords:** MASLD, lipid droplets, carboxylesterase, triacylglycerol synthesis, DGAT, LXR, SREBP1c, oxysterol, PPARγ

## Abstract

Increased hepatic triacylglycerol (TG) storage in lipid droplets (LDs) is a hallmark of metabolic dysfunction-associated steatotic liver disease (MASLD) and metabolic dysfunction-associated steatohepatitis (MASH). Human carboxylesterase 1 (CES1) regulates TG storage and secretion in hepatocytes, but the mechanism remains to be elucidated. We performed studies in rat hepatoma McArdle RH7777 cells stably transfected with CES1 cDNA and in Ces1d-deficient mice using a variety of biochemical, pharmacological and cell biology approaches including the assessment of gene expression, confocal immunofluorescence microscopy, lipid synthesis measurements and quantitative mass spectrometry. CES1-expressing cells accrued more TG compared to cells lacking CES1 when incubated with oleic acid. CES1 increased the expression of *Srebf1c*, *Nr1h3* and *Nr1h2* encoding transcription factors (SREBP1c and LXRα and LXRβ, respectively) that regulate the expression of lipogenic genes. Additionally, CES1 increased the expression of *Acsl1* encoding an enzyme catalyzing fatty acid activation and the expression of *Dgat1* and *Dgat2* encoding enzymes catalyzing TG synthesis. Treatment of CES1-expressing cells with PPARγ antagonist (GW9662), LXR antagonist (GSK2033) or CYP27A1 inhibitor Felodipine prevented CES1-mediated fatty acid esterification into TG. Ces1d-deficient mice fed high-fat diet (HFD) presented with decreased expression of *Nr1h3, Nr1h2*, *Srebf1c* and reduced hepatic TG content. Felodipine and GSK2033 treatment eliminated the differential effects on TG concentration between wild-type and Ces1d-deficient hepatocytes. The results suggest that CES1/Ces1d activates PPARγ, LXR and SREBP1c pathways, thereby increasing TG synthesis and LD storage by augmenting fatty acid esterification.

## 1. Introduction

Increased hepatic triacylglycerol (TG) synthesis and storage in cytosolic lipid droplets (LDs) is a hallmark of metabolic dysfunction-associated steatotic liver disease (MASLD) [1,2,3] and is strongly associated with insulin resistance and diabetes [4]. Hepatic TG accumulation can occur through increased TG synthesis, decreased very low-density lipoprotein (VLDL) secretion, or reduced fatty acid (FA) oxidation [5]. In MASLD, lipolysis of adipose tissue fat stores provides about 60% of FA used for hepatic TG synthesis, whereas up to 25% can be obtained from de novo lipogenesis and the remaining 15% is derived from dietary sources [5,6,7].

Several studies have demonstrated a role for endoplasmic reticulum-localized carboxylesterases in mouse and human hepatic TG metabolism (reviewed in [8]). Elevation in carboxylesterase 1 (*CES1*) gene expression has been observed in patients with MASLD [9], while genetic variants in *CES1* gene that decrease CES1 lipolytic activity improved hyperlipidemia and steatosis in an HFD-fed humanized mouse model. The mouse ortholog of CES1 is carboxylesterase 1d (Ces1d), previously also annotated as Ces3 or TGH [8]. Genetic ablation of *Ces1d* expression resulted in beneficial phenotypes in mice fed chow, high-fat diet or Western-type diet including reduced hepatic and plasma TG concentrations, increased FA β-oxidation and insulin sensitivity [10,11]. Accumulating evidence suggests that CES1/Ces1d alters the morphology of lipid droplets (LDs). Hepatocytes (over)expressing CES1/Ces1d store TG in larger LDs, whereas hepatocytes lacking Ces1d or expressing catalytically impaired CES1 form small LDs [12,13]. Decreased TG lipase activity is typically associated with increased TG storage, so the reduction in hepatic TG storage, upon eliminating CES1/Ces1d activity and increased TG storage in cells expressing active CES1/Ces1d, was unexpected. The mechanism through which CES1/Ces1d lipolytic activity increases TG storage in larger LDs has not yet been elucidated.

To address the mechanism of CES1/Ces1d regulation of TG metabolism, we have interrogated TG synthesis and storage in rat hepatoma McArdle RH7777 cells stably expressing CES1 cDNA and in primary hepatocytes isolated from Ces1d-deficient mice. We found that CES1 lipase activity increases expression and/or activity of key lipogenic transcription factors PPARγ, LXR and SREBP1c and consequently the expression of genes encoding enzymes involved in TG synthesis and storage.

## 2. Materials and Methods

### 2.1. Materials

Dulbecco’s Modified Eagle’s Medium (DMEM), fetal bovine serum (FBS), and horse serum (HS) were purchased from Invitrogen Canada (Burlington, ON, Canada). Fatty acid-free bovine serum albumin (BSA) and Complete protease inhibitor cocktail tablets were procured from Roche Diagnostics (Laval, QC, Canada). BODIPY 493/503 and BODIPY FL C12 were purchased from Invitrogen (Carlsbad, CA, USA). [9,10(n)-^3^H]Oleic Acid (OA) (54.6 Ci/mmol) was from Perkin Elmer (Waltham, MA, USA); OA, inhibitors of DGAT1 (PF-04620110) and DGAT2 (PF-06424439), LXR antagonist GSK2033, PPARγ antagonist GW9662 and CYP27A1 inhibitor Felodipine were obtained from Sigma-Aldrich (Oakville, ON, Canada). All other reagents were of analytical grade or higher.

### 2.2. Mice

All animal experiments were approved by the University of Alberta Animal Care and Use Committee and were performed following the guidelines of the Canadian Council on Animal Care. *Ces1d^-/-^* mice generated previously [11] were backcrossed into the C57BL/6J background for 10 generations. Mice were maintained on a chow diet (LabDiet, PICO laboratory Rodent diet 20). At 10 weeks of age, age-matched male C57BL/6J and *Ces1d^-/-^* mice were fed a high-fat diet (HFD, F3282, Bioserv, Flemington, NJ, USA) containing 60% calories from fat for 16 weeks. Livers were collected from 5 h fasted mice.

### 2.3. Preparation of Primary Mouse Hepatocytes

Primary mouse hepatocytes were isolated by collagenase perfusion of livers from chow fed wild-type (C57BL/6J) and *Ces1d^-/-^* mice and plated on 60 mm collagen-coated dishes at a confluence of 1.0 × 10^6^ cells/dish, or on #1 coverslips (BD BioCoat^TM^, Thermo Fisher, Waltham, MA, USA) in six-well plates at 2.0 × 10^5^ cells/well. Hepatocytes were allowed to attach in DMEM supplemented with 15% FBS at 37 °C in humidified air containing 5% CO_2_ for 4 h, followed by incubations with experimental media.

### 2.4. Cell Culture

Rat hepatoma McArdle RH7777 cells were obtained from American Type Culture Collection (Manassas, VA, USA). McArdle RH7777 cells stably transfected with either empty pCi Neo vector (control pNeo cells) or with pCi Neo vector containing CES1 cDNA (CES1 cells) were produced in our laboratory [13]. Cells were maintained in DMEM containing 10% fetal bovine serum (*v*/*v*) and 10% HS (*v*/*v*) and incubated at 37 °C in an atmosphere enriched with 5% CO_2_ in the presence of 50 units/mL penicillin and 50 µg/mL streptomycin and 0.4 mg/mL G-418.

### 2.5. High-Performance Liquid Chromatography (HPLC) Analysis of Lipids

HPLC was used to quantify the mass of TG, cholesteryl esters (CE), unesterified cholesterol and phospholipids. The analysis was carried out on the Agilent 1100 instrument equipped with a quaternary pump and Alltech ELSD2000 Evaporative Light-Scattering Detector (Grace Davision/Alltech, Columbia, MA, USA), using a modified version of the method of Graeve and Janssen [14]. In brief, protein concentration in cell homogenates was determined using the Bradford method and lipids were extracted from homogenates equivalent to 1 mg protein by modified Folch method [15] in the presence of 30 µg batyl alcohol (glycerol-1-stearyl-ether) as the internal standard. The lipid-containing chloroform phase was removed and dried under a stream of nitrogen, resuspended in 100 µL chloroform: isooctane (1:1) and 5 µL was injected onto an Onyx monolithic silica normal-phase column (Phenomenex, Torrance, CA, USA). Lipids were separated using a three-solvent gradient with a flow rate of 1.4 mL/min.

### 2.6. Metabolic Studies

pNeo and CES1 cells were pre-incubated for 1 h in serum-free medium with or without 5 µM DGAT1, DGAT2 or both DGAT1 and DGAT2 inhibitors. This was followed by a 12 h pulse incubation with 2.5 µCi/mL [^3^H]OA and 0.4 mM OA complexed with 0.5% fatty acid-free BSA in the presence or absence of DGAT1 or DGAT2 or both DGAT1 and DGAT2 inhibitors (5 µM concentration of each inhibitor)]. A similar protocol was followed using Felodipine (30 µM), GSK2033 (10 µM) and GW9662 (10 µM) incubations of McArdle RH7777 cells and primary mouse hepatocytes. Cells were harvested and lipids were extracted using chloroform–methanol (2:1, *v*/*v*) and resolved by thin-layer chromatography (TLC) using heptane/isopropyl ether/acetic acid (15:10:1 by volume). Radioactivity in various lipids was quantified by a Bioscan radio-TLC Imaging Scanner (AR-2000) (Eckert & Ziegler Medical, Berlin, Germany) with the Winscan software system (Version 3.14, Eckert & Ziegler Radiopharma, Hopkinton, MA, USA) and by scintillation counting.

### 2.7. Gene Expression Analyses

pNeo and CES1 cells were pre-incubated for 1 h in serum-free medium in the presence/absence of the indicated inhibitors, followed by a 12 h incubation in DMEM + 0.4 mM OA complexed with 0.5% fatty acid-free BSA in the presence/absence of the inhibitors. Total RNA from cells or mouse livers was isolated using TRIZOL Reagent (Invitrogen, Carlsbad, CA, USA) according to the manufacturer’s instructions. First strand cDNA was synthesized from 2 μg of total RNA using Superscript ΙΙΙ reverse transcriptase (Invitrogen) primed by oligo (dt)12-18 (Invitrogen) and random primers (Invitrogen). Real-time quantitative qPCR was performed with Power SYBR^®^ Green PCR Master Mix kit (Life Technologies/Thermo Fisher, Carlsbad, CA, USA) using the StepOnePlus real-time PCR system (Applied Biosystems,/Thermo Fisher, Carlsbad, CA, USA). All primers were synthesized by Integrated DNA Technologies (Coralville, IA, USA). Primers for genes tested are listed in Supplemental Table S1. Data were analyzed with the StepOne software (Applied Biosystems/Thermo Fisher, Carlsbad, CA, USA). Standard curves were used to calculate mRNA abundance relative to that of the control gene *Ppia* encoding cyclophilin A.

### 2.8. Immunoblotting

Cells were harvested in ice-cold IM buffer (250 mM sucrose, 50 mM Tris, 1 mM EDTA, pH 7.4), sonicated and protein concentration was determined. Proteins were separated by SDS-PAGE, transferred to Immobilon-P transfer membrane (PVDF) (catalog #IPVH00010; Millipore) and subjected to immunoblotting. The following primary antibodies (diluted in 3% *w*/*v* BSA in TBST) were used: acyl-CoA synthetase/ligase 1 (ACSL1) (1:1000, Cell Signaling, #4047), perilipin 2 (PLIN2) (1:1000, Abcam #108323), CYP27A1 (1:1000, Abcam, #126785), β-Actin (1:1000, Cell Signaling, #4967), glyceraldehyde 3-phosphate dehydrogenase (GAPDH) (1:5000, Abcam #ab8245). Membranes were incubated with primary antibodies overnight (12-16 h) at 4 °C. After washing, the membranes were incubated with appropriate secondary antibodies, diluted to 1:5000 in 5% *w*/*v* skimmed milk in TBST, for 1 h at room temperature. The secondary antibodies were HRP-labeled goat anti-rabbit IgG (1:5000, Invitrogen, #31460) and HRP-labeled goat anti-mouse IgG (1:5000, Invitrogen, #31460). Immunoreactive proteins were detected by enhanced chemiluminescence (Immobilon-Millipore, Oakville, ON, Canada, #WBCUC0500) and visualized by G:BOX Chemi XX8 (SynGene, Cambridge, UK). The resulting bands relative intensities on the blots were quantified by densitometry using the GeneTools program (SynGene, Cambridge, UK).

### 2.9. Analysis of LDs by Confocal Fluorescence Scanning Microscopy

pNeo and CES1 cells were incubated for 1 h in serum-free medium followed by 12 h incubation in DMEM containing 0.4 mM OA complexed to 0.5% fatty acid-free BSA. Following incubation, cells were washed three times with phosphate-buffered saline (PBS), fixed with 4% formaldehyde for 20 min, followed by three washes with PBS. LDs were labeled with 2 μg/mL BODIPY 493/503 in PBS for 30 min. To visualize new LD formation, cells were pre-incubated for 1 h in serum-free medium with or without indicated inhibitors followed by 12 h incubation in DMEM containing 0.4 mM OA and 6 μM BODIPY FL C12 complexed to 0.5% fatty acid-free BSA. Samples were mounted to slides using Vectashield mounting medium for fluorescence with DAPI (Vector Laboratories Inc., Newark, CA, USA, Cat# H-1200). Images were captured by a spinning disk confocal microscope (Olympus IX81-DSU, Tokyo, Japan). The quantification of LD numbers and sizes were determined using Fiji-ImageJ.

### 2.10. Oxysterol Determination by LC-MS/MS

Free and esterified oxysterols were measured as previously described [16]. Briefly, 100 mg of liver was homogenized in 4 mL chloroform–methanol (2:1, *v*/*v*) containing 50 μg/mL butylated hydroxytoluene and 100 pmol of each internal standard (24(R/S)-Hydroxycholesterol-d7, 25-Hydroxycholesterol-d6, 25(R/S), 26-Hydroxycholesterol-d4; C/D/N Isotopes, Quebec, Canada). The sample was split in half, with one half used for free oxysterol measurement, and the other half saponified for total oxysterol measurement. Oxysterols were subsequently extracted by solid phase extraction using 100 mg Silica SPE columns (waters). Samples were dried under a constant stream of N_2_ and reconstituted in 75 μL of methanol for analysis by LC-MS/MS. Esterified oxysterols were determined by subtracting the free oxysterols from total oxysterols.

### 2.11. Statistical Analysis

Data are plotted as mean ± SEM. Significant differences between two groups were determined by unpaired two-tailed *t*-tests. Studies using pNeo and CES1 cells represent at least 3 independent biological experiments, each performed in triplicate. Data were analyzed by two-way ANOVA followed by Bonferroni post hoc tests (Graph Pad PRISM 8 software, Boston, MA, USA). Differences were considered statistically significant at * *p* < 0.05, ** *p* < 0.01, *** *p* < 0.001 and **** *p* < 0.0001.

## 3. Results

### 3.1. CES1 Facilitates TG Accumulation and Large LD Formation

**Figure 1:**
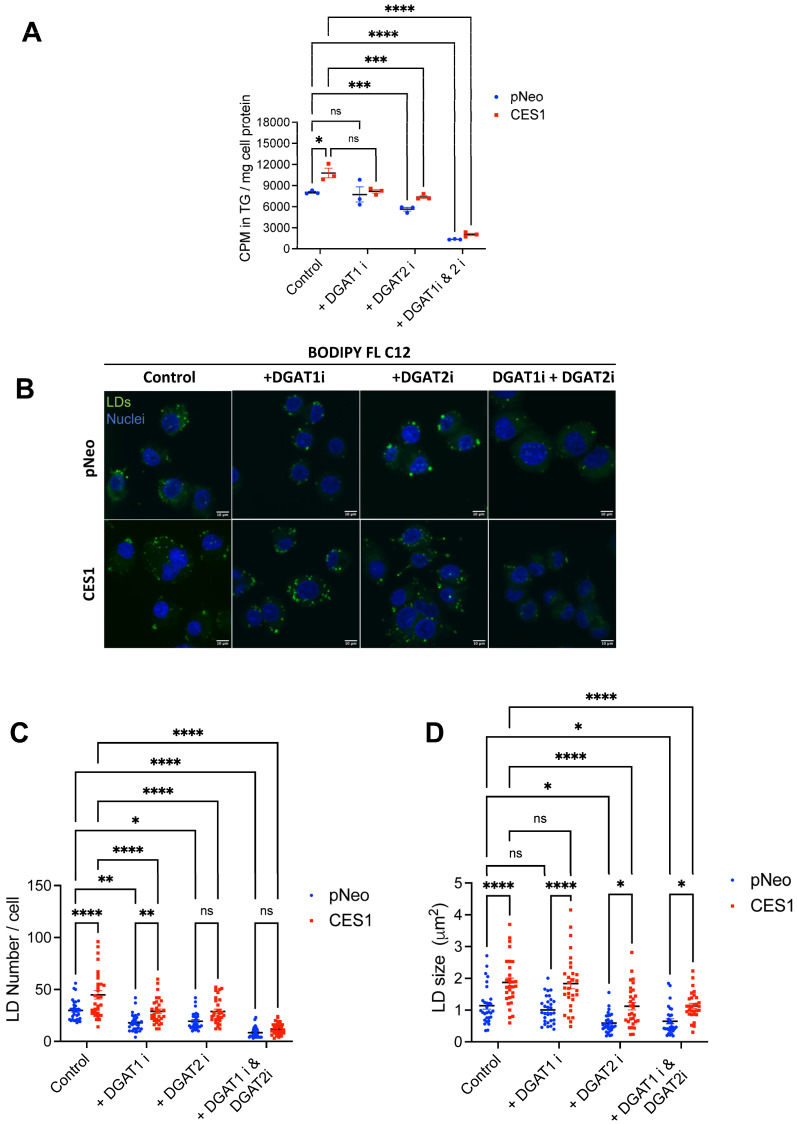
CES1 promotes TG synthesis that is dependent on both DGAT1 and DGAT2 activities. (**A**) pNeo and CES1 cells were incubated with 0.4 mM [^3^H]OA complexed to 0.5% BSA in the presence or absence of 5 µM DGAT inhibitors for 12 h, and radioactivity in TG was determined following separation of lipids by thin-layer chromatography. (**B**) Newly synthesized LDs visualized in pNeo and CES1 cells after 12 h incubation with 6 µM BODIPY FL C12 fatty acid and 0.4 mM OA complexed with 0.5% BSA. Scale bars = 10 µm. (**C**) The number of newly synthesized LDs. LD analysis was performed in 30 cells of each group. (**D**) The size of newly synthesized LDs. LD analysis was performed in 30 cells of each group. ns: not significant, * *p* < 0.05, ** *p* < 0.01, *** *p* < 0.001, and **** *p* < 0.0001.

Our previous findings showed that CES1/Ces1d participates in hepatic lipid homeostasis and VLDL assembly [10,11]. Interestingly, a higher number of smaller LDs was observed in hepatocytes lacking Ces1d/CES1 expression [13]. Re-expression of active CES1, but not catalytically dead CES1, in the livers of *Ces1d^-/-^* mice reversed the LD phenotype, indicating that CES1 lipolytic activity modulates LD size and number [13]; however, the mechanism is unknown. To address whether CES1 promotes FA incorporation into neutral lipids, we used McArdle RH7777 hepatocytes that do not express endogenous Ces1d stably transfected with either an empty pCI Neo vector (control pNeo cells) or a pCI Neo vector containing CES1 cDNA (CES1 cells) (Figure S1 and [13]). CES1 cells exhibit significantly augmented esterase activity compared to pNeo cells [13]. The level of expression of CES1 in CES1 cells is lower than observed in human liver (Figure S1). After incubation of pNeo and CES1 cells with OA we observed a ~30% increase in the incorporation of OA into TG in CES1 cells compared to pNeo cells (Figure 1A). An increased trend of OA incorporation into cholesteryl esters (CE) in CES1 cells was also observed (Figure S2A). Incubation of CES1 cells with OA also augmented OA incorporation into phosphatidylcholine (PC) but not into phosphatidylethanolamine (PE) or phosphatidylinositol (PI) (Figure S2B). OA incubation resulted in an increased number and larger size LDs in CES1 cells (Figure S2C-E).

In the final step of the synthesis of TG, acyl-CoA:diacylglycerol-O-acyltransferases 1 and 2 (DGAT1 and DGAT2) esterify diacylglycerol (DG) to TG [17,18,19,20]. To investigate whether CES1 promotes TG synthesis via DGAT1 or DGAT2, we incubated pNeo and CES1 cells with small molecule inhibitors of DGAT1 and DGAT2, either individually or together, and assessed incorporation of OA into TG. DGAT2 fully compensated for the loss of DGAT1 activity to support TG synthesis in pNeo cells and there was a trend towards decreased TG synthesis in CES1 cells, suggesting that some of the CES1-mediated increase in TG synthesis could be potentially catalyzed by DGAT1 (Figure 1A). Inhibition of DGAT2 resulted in approximately a 40% decrease in TG synthesis in both pNeo and CES1 cells (Figure 1A). Combined inhibition nearly completely prevented TG synthesis in both pNeo and CES1 cells, confirming the efficacy of the inhibitors and demonstrating that DGAT1 and DGAT2 are the sole enzymes responsible for fatty acid esterification into TG in these cells (Figure 1A).

Increased OA-driven TG synthesis in CES1 cells was accompanied by an increased number and size of LDs (Figure 1B–D). These results suggest that CES1 promotes LD formation through facilitation of fatty acid esterification into TG and PC. Inhibition of DGAT1 resulted in a significant reduction in the number of LDs in both pNeo and CES1 cells, but there was no normalization of LD numbers between pNeo and CES1 cells (Figure 1B,C). Inhibition of DGAT2 also decreased LD numbers in both pNeo and CES1 cells. DGAT2 inhibition normalized LD numbers between the two cell lines. DGAT1 inhibition did not influence the size of LDs in either pNeo or CES1 cells, while inhibition of DGAT2 or both DGAT1 and DGAT2 resulted in smaller size LDs in both pNeo and CES1 cells (Figure 1D). These findings suggest that both DGAT1 and DGAT2 contribute to CES1-mediated TG accretion in LDs.

### 3.2. CES1 Enhances Expression of Genes Encoding Lipogenic Transcription Factors and Enzymes Catalyzing Fatty Acid Activation and Neutral Lipid Synthesis

**Figure 2:**
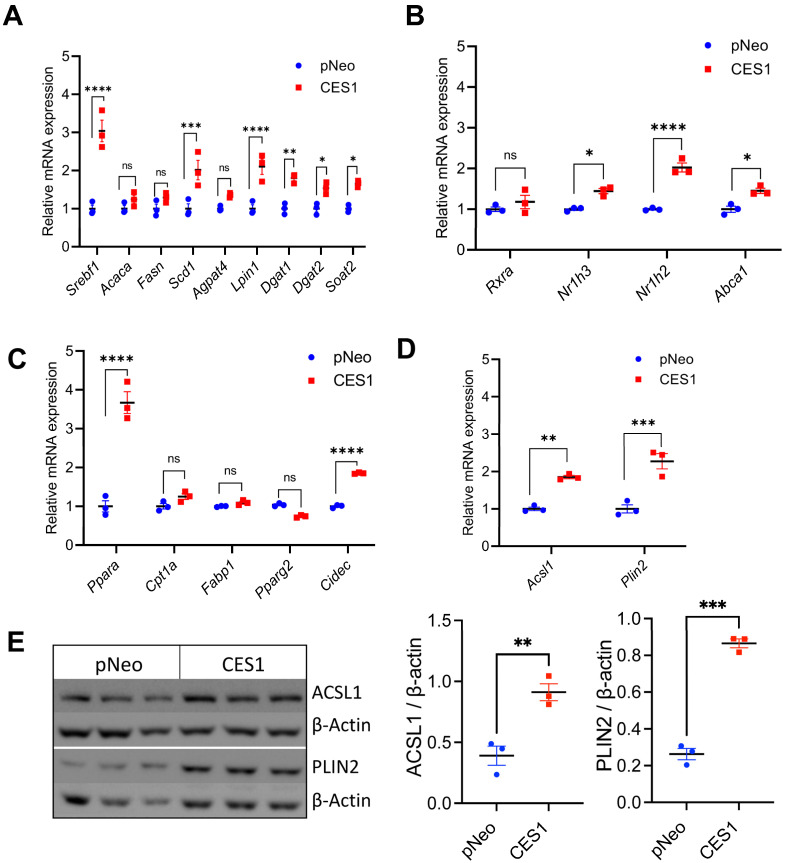
CES1 increases expression of genes encoding TG synthesis and storage regulators. pNeo and CES1 cells were incubated with 0.4 mM OA complexed with 0.5% BSA for 12 h. mRNA was isolated and expression of genes encoding proteins/enzymes/regulators involved in neutral lipid synthesis was determined by qPCR analysis. Results are presented as the ratio of expression of a given gene to cyclophilin with the expression in pNeo cells set at a value of 1. Protein expression was determined by immunoblotting. Data are means ± SEM of three independent experiments performed in triplicate. ns: not significant, * *p* < 0.05, ** *p* < 0.01, *** *p* < 0.001 and **** *p* < 0.0001. (**A**) Relative mRNA expression of *Srebf1c*, *Acaca*, *Fasn, Scd* and genes encoding enzymes involved in fatty acid esterification *Agpat4, Lpin1, Dgat1, Dgat2* and *Soat2*. (**B**) Relative mRNA expression of *Nr1h3* and *Nr1h2*, and the LXR target gene *Abca1*. (**C**) Relative expression of *Ppara* and *Pparg,* and PPARα target genes *Cpt1a*, *Fabp1* and PPARα/γ target gene *Cidec*. (**D**) Relative mRNA expression of *Acsl1 and Plin2*. (**E**) Immunoblot analysis of ACSL1 and LD coat protein PLIN2. Immunoblotting of b-Actin was used as a loading control.

To further delineate the mechanism of increased neutral lipid storage in CES1 cells, we analyzed the expression of genes encoding regulators and enzymes participating in neutral lipid synthesis. The key lipogenic transcriptional regulators are SREBP1c, LXRα and LXRβ encoded by *Srebf1*, *Nr1h3* and *Nr1h2* genes, respectively. Expression of *Srebf1* and the SREBP1c target gene *Scd* encoding stearoyl-CoA desaturase was significantly increased in CES1 cells (Figure 2A). Expression of *Nr1h3, Nr1h2* and the LXR target gene *Abca1* was also augmented in CES1 cells (Figure 2B). Additionally, expression of several genes encoding critical enzymes in neutral lipid synthesis was increased in CES1 cells including *Lpin1*, *Dgat1*, *Dgat2* and *Soat2* (encoding acyl-CoA:cholesterol acyltransferase 2, ACAT2) (Figure 2A). However, expression of *Acaca* and *Fasn* and encoded enzymes acetyl-CoA carboxylase (ACC1) and fatty acid synthase (FASN), the key enzymes in *de novo* fatty acid synthesis, were similar in pNeo and CES1 cells (Figure 2A and Figure S3). Expression of *Ppara* but not *Pparg* was upregulated in CES1 cells (Figure 2C), though upregulation of PPAR expression itself is not sufficient since PPARs require the presence of fatty acid-derived ligands for activation. Expression of PPARγ target gene *Cidec* was upregulated in CES1 cells (Figure 2C), suggesting increased activation of PPARγ signaling. PPARs also regulate the transcription of *Acsl1* and *Plin2* genes [21,22,23]. *Acsl1* encodes a long-chain acyl-CoA synthetase (ACSL) required for the activation of fatty acids [24], while *Plin2* encodes a canonical LD binding protein PLIN2 that participates in the regulation of LD turnover and is often used as a marker of LD abundance. CES1 cells have higher abundance of *Acsl1* and *Plin2* mRNA as well as ACSL1 and PLIN2 proteins compared to pNeo cells (Figure 2D,E). 

**Figure 3:**
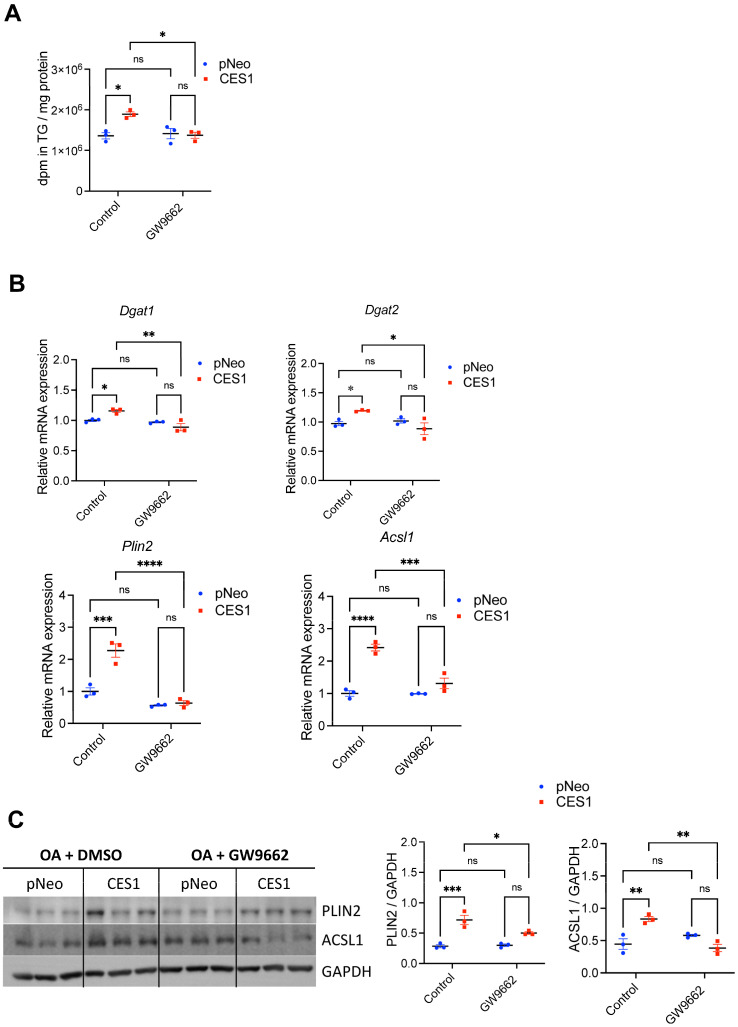
CES1 enhances TG synthesis through a PPARγ pathway. pNeo and CES1 cells were incubated with 0.4 mM [^3^H]OA complexed with 0.5% BSA for 12 h ± PPARγ antagonist GW9662 and incorporation of OA into TG was determined following lipid extraction and TLC using Bioscan radio-TLC Imaging Scanner. Cells incubated with non-labeled OA ± GW9662 were used for mRNA isolation and for protein analysis. Results are presented as the ratio of expression of a given gene to cyclophilin with the expression in pNeo cells set at a value of 1. Protein expression was determined by immunoblotting. Data are means ± SEM of three independent experiments performed in triplicate. ns, not significant, * *p* < 0.05, ** *p* < 0.01, *** *p* < 0.001 and **** *p* < 0.0001. (**A**) TG synthesis from [^3^H]OA in the presence or absence of PPARγ antagonist GW9662. (**B**) mRNA expression of *Dgat1, Dgat2, Acsl1* and *Plin2* in pNeo and CES1 cells ± treatment with PPARγ antagonist GW9662. (**C**) Immunoblot analysis of PLIN2 and ACSL1 in pNeo and CES1 cells ± treatment with PPARγ antagonist GW9662. GAPDH immunoblotting was used as a loading control.

To elucidate whether CES1-dependent TG accretion is mediated via PPARγ we treated the cells with the PPARγ antagonist GW9662. GW9662 decreased CES1-mediated TG synthesis (Figure 3A) and the expression of TG synthesis and storage genes (Figure 3B) and proteins (Figure 3C) but had no effect on phospholipid synthesis (Figure S4A). These results suggest that some of the effect of CES1 on neutral lipid synthesis and storage could be in part due to regulation of PPARγ activity.

### 3.3. CYP27A1 and LXR Inhibitors Attenuate LXR Transcriptional Regulation in CES1 Cells

**Figure 4:**
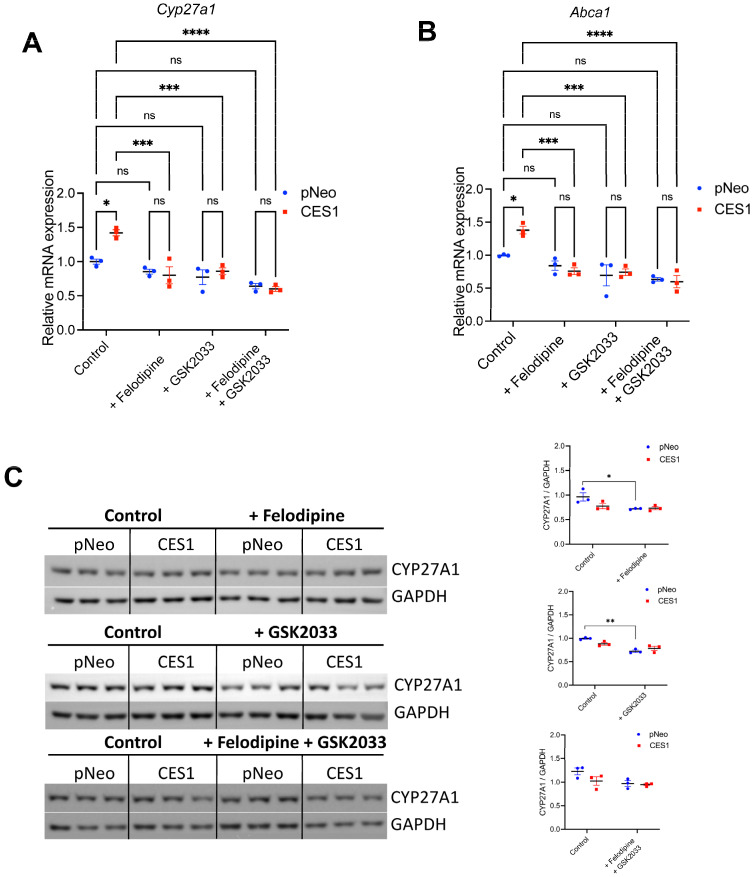
Inactivation of CYP27A1 and LXR attenuates LXR mediated transcriptional regulation in CES1 cells. pNeo and CES1 cells were incubated with 0.4 mM OA complexed with 0.5% BSA for 12 h ± CYP27A1 inhibitor Felodipine, LXR antagonist GSK2033 or a combination of Felodipine and GSK20233. mRNA was isolated and expression of genes encoding CYP27A1 and LXR target gene *Abca1* were determined by qPCR analysis. Results are presented as the ratio of expression of a given gene to cyclophilin with the expression in pNeo cells set at a value of 1. Protein expression was determined by immunoblotting. Data are means ± SEM of three independent experiments performed in triplicate. ns, not significant, * *p* < 0.05, ** *p* < 0.01, *** *p* < 0.001 and **** *p* < 0.0001. (**A**) Expression *Cyp27a1* mRNA. (**B**) Expression *Abca1* mRNA. (**C**) Expression of CYP27A1 protein.

Expression of genes encoding lipogenic regulators LXRα and LXRβ and LXRα/β target gene *Abca1* was upregulated in CES1 cells (Figure 2B). The expression of another LXR target gene *Cyp27a1* was also upregulated in CES1 cells (Figure 4A). Ablation of *CES1* expression in THP-1 cells reduced the production of 27-hydroxycholesterol (27-HC) via CYP27A1 [25]. This oxysterol is a potent ligand and activator of LXR [26,27,28], and therefore, we hypothesized that increased OA esterification to TG and increased TG storage in CES1 cells could be additionally due to augmented CYP27A1-mediated activation of the LXR pathway. To test this hypothesis, we employed a specific inhibitor of CYP27A1 Felodipine [29] and the LXR antagonist GSK2033. Felodipine significantly reduced CES1-mediated expression of *Cyp27a1* and LXR target gene *Abca1* to control pNeo cell levels (Figure 4A,B). We also determined the effects of Felodipine and GSK2033 on CYP27A1 protein expression. Surprisingly, there was no statistical difference between pNeo and CES1 cells in CYP27A1 protein abundance, while individual treatments with Felodipine or GSK2033 slightly decreased CYP27A1 abundance in pNeo cells but not in CES1 cells (Figure 4C). These results suggest that CES1 expression increased *Cyp27a1* and *Abca1* mRNA abundance without altering CYP27A1 protein levels.

### 3.4. Inhibition of CYP27A1 and LXR Reduces TG Accumulation in CES1 Cells

**Figure 5:**
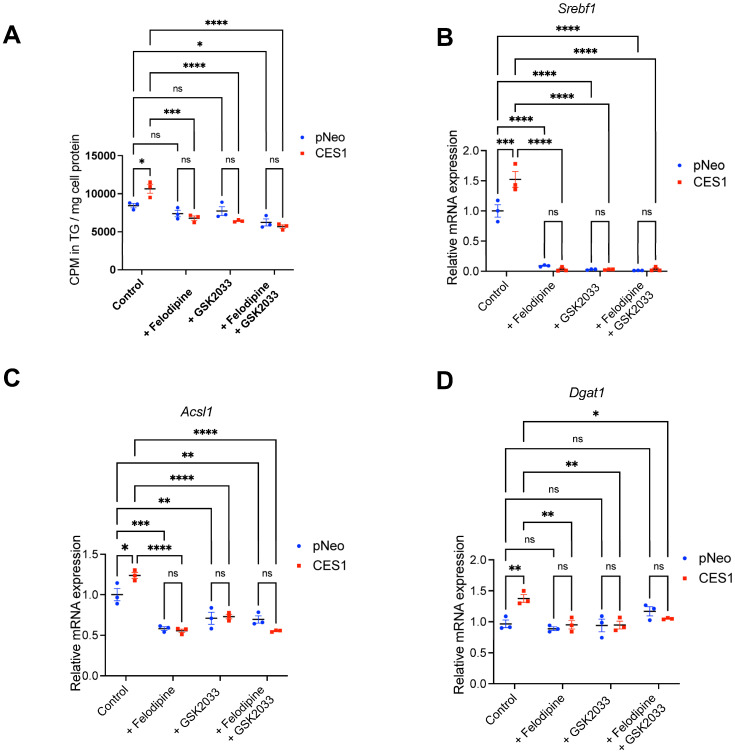

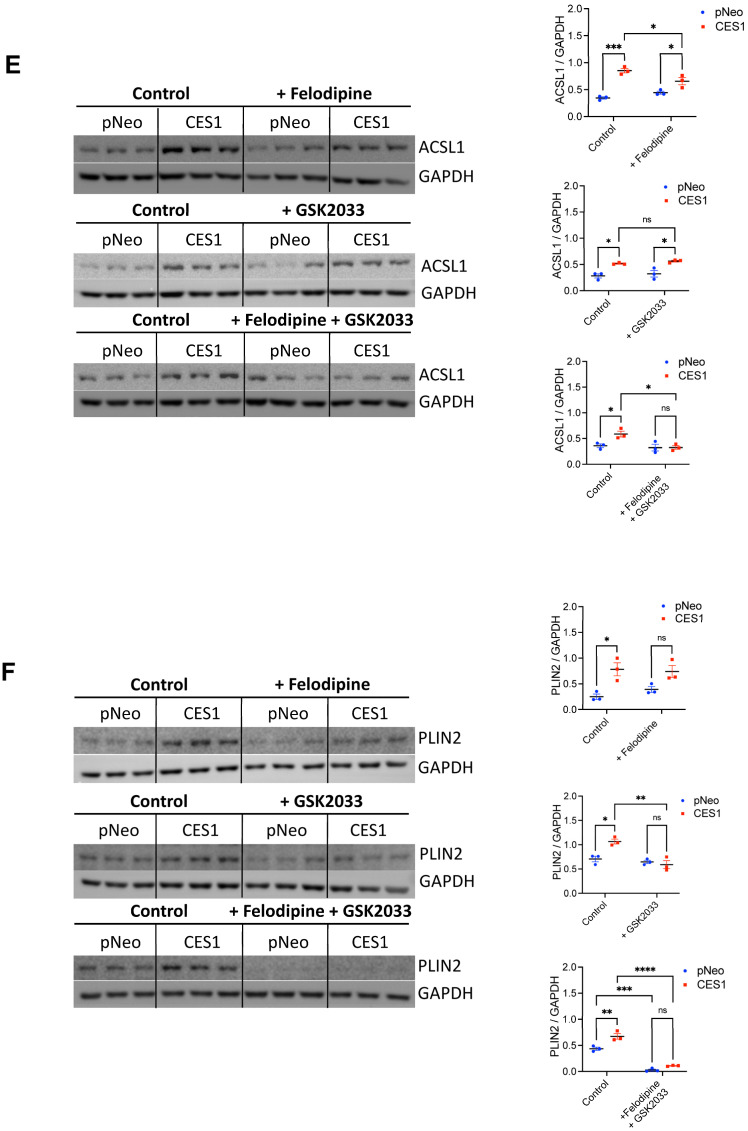
Inhibition of CYP27A1 and LXR reduces CES1-mediated TG synthesis. pNeo and CES1 cells were incubated with 0.4 mM [^3^H]OA complexed with 0.5% BSA for 12 h ± CYP27A1 inhibitor Felodipine, LXR antagonist GSK2033 or both Felodipine and GSK2023 together and incorporation of OA into TG was determined following lipid extraction and TLC using Bioscan radio-TLC Imaging Scanner. Cells incubated with non-labeled OA ± Felodipine, GSK2023 or both Felodipine and GSK2023 were used for mRNA isolation and for protein analysis. Results are presented as the ratio of expression of a given gene to cyclophilin with the expression in pNeo cells set at a value of 1. Protein expression was determined by immunoblotting. Data are means ± SEM of three independent experiments performed in triplicate. ns, not significant, * *p* < 0.05, ** *p* < 0.01, *** *p* < 0.001 and **** *p* < 0.0001. TG synthesis from [^3^H]OA in the presence or absence of 10 μM GSK2033 or 30 μM Felodipine alone or in combination. (**A**) TG synthesis. (**B**) *Srebf1* expression. (**C**) *Acsl1* expression. (**D**) *Dgat1* expression. (**E**) ACSL1 protein expression. (**F**) PLIN2 protein expression.

Inhibition of CYP27A1 or LXR decreased CES1-mediated increase in TG synthesis (Figure 5A). Phospholipid synthesis did not appear to be affected by either inhibitor but was decreased in both CES1 and pNeo cells with combined CYP27A1 and LXR inhibition (Figure S4B). Inactivation of CYP27A1 or LXR dramatically reduced *Srebf1* mRNA expression in both pNeo and CES1 cells (Figure 5B) and decreased *Acsl1* expression (Figure 5C), and the treatment also reduced *Dgat1* expression in CES1 cells but not in pNeo cells (Figure 5D). The inhibitors had no effect on *Dgat2* expression.

ACSL1 protein abundance was slightly reduced in CES1 cells with CYP27A1 inhibition or with combined CYP27A1 and LXR inhibition (Figure 5E). CYP27A1 inhibition did not appear to significantly affect PLIN2 abundance in either pNeo or CES1 cells (Figure 5F). Treatment with the LXR antagonist reduced PLIN2 abundance in CES1 cells, and a combination of both LXR and CYP27A1 inhibitors dramatically decreased PLIN2 abundance in both cell lines (Figure 5F).

### 3.5. Ces1d Deficiency Reduces Neutral Lipid Synthesis and Srebf1c, Nr1h2, Nrh1h3, and Rxra Expression

**Figure 6:**
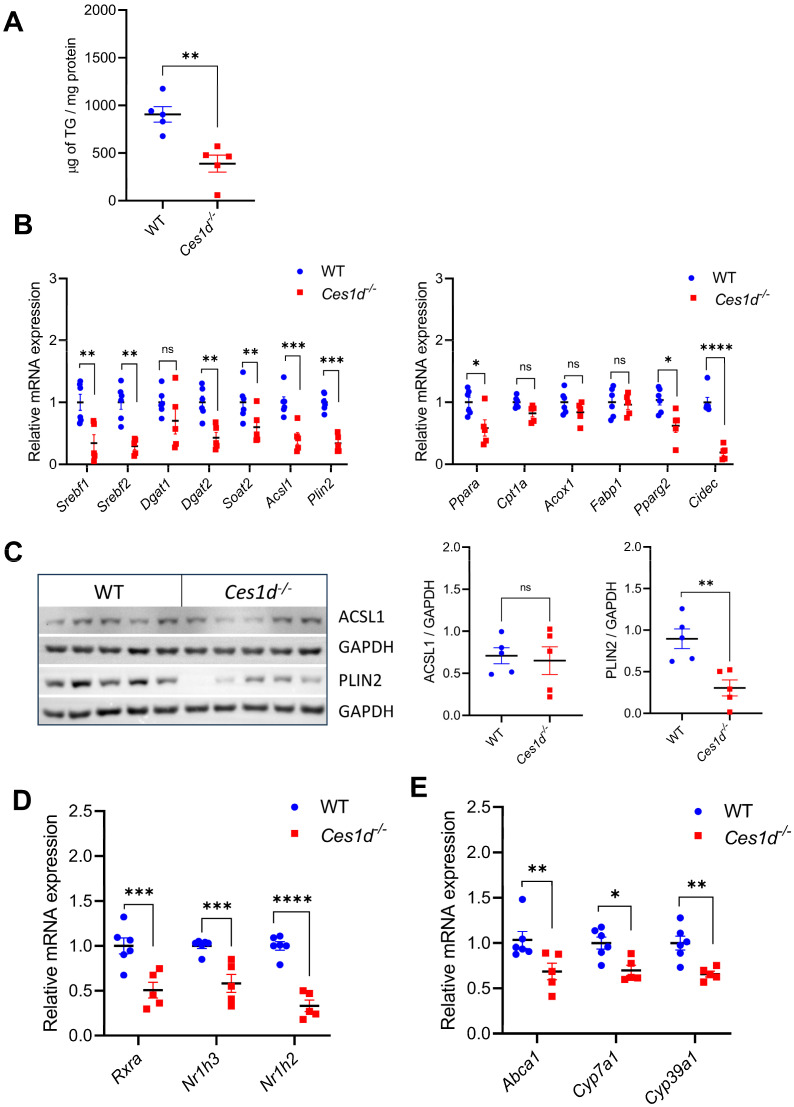

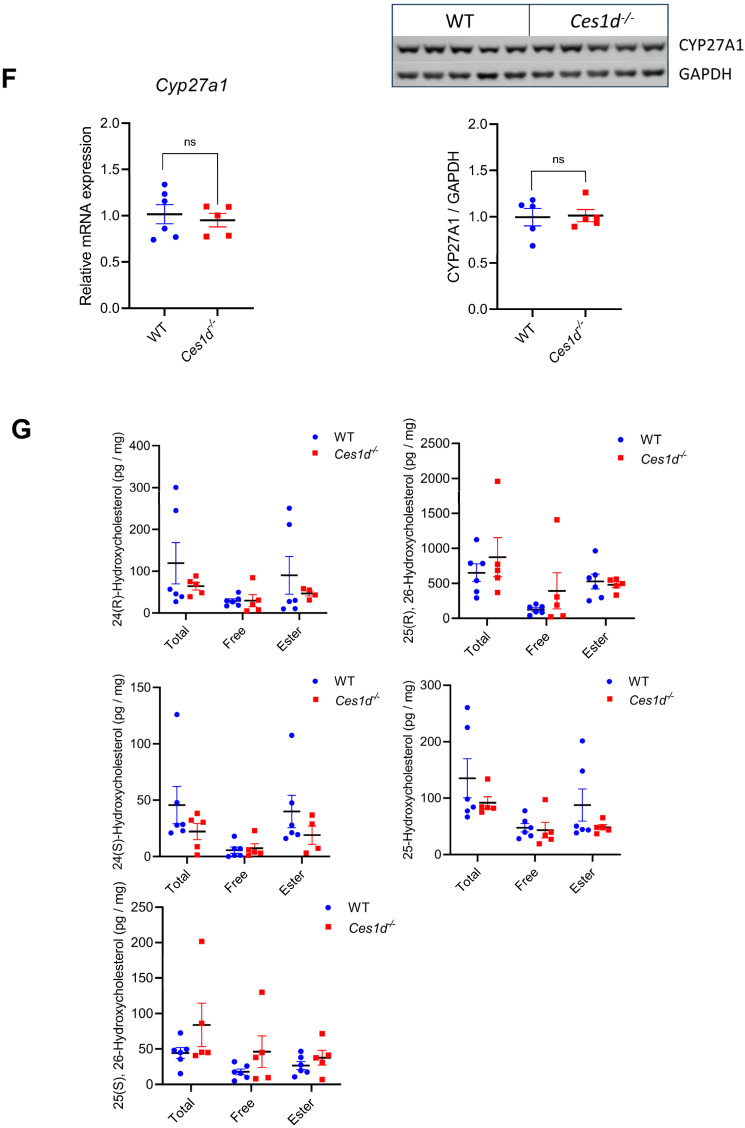
Ces1d deficiency results in decreased hepatic TG storage and expression of genes encoding transcription factors and enzymes responsible for neutral lipid synthesis and storage. Male WT (C57BL/6J) and *Ces1d^−/−^* mice (n = 5) were fed HFD for 16 weeks from the age of 4 weeks. Data are means ± SEM. ns, not significant, * *p* < 0.05, ** *p* < 0.01, *** *p* < 0.001 and **** *p* < 0.0001. (**A**) Hepatic TG concentration determined by HPLC. (**B**) Hepatic mRNA abundance of genes encoding transcription factors, proteins and enzymes regulating neutral lipid synthesis, storage and oxidation determined by qPCR. (**C**) Hepatic ACSL1 and LD coat protein PLIN2 expression in HFD-fed WT and *Ces1d*^−/−^ mice was assessed by immunoblotting. Immunoblotting of GAPDH served as a loading control. (**D**) Hepatic mRNA abundance of genes encoding transcription factors RXR and LXRα/β. (**E**) Hepatic mRNA abundance of LXR target genes *Abca1*, *Cyp7a1* and *Cyp39a1.* **(F**) Abundance of hepatic *Cyp27a1* mRNA and CYP27A1 protein. Immunoblotting of GAPDH served as a loading control. (**G**) Hepatic concentrations of total, free and ester forms of oxysterols determined by LC-MS/MS.

Deficiency in the CES1 ortholog Ces1d expression in mice results in a beneficial phenotype, including protection against HFD-induced hepatic steatosis [10]. However, the mechanism of this protection is not fully understood. In this context, we examined the role of Ces1d in activating PPARγ-LXR-SREBP1-mediated neutral lipid synthetic pathways in mice fed HFD. HFD-fed *Ces1d^-/-^* mice exhibited a reduction in hepatic TG content (~40%) when compared with WT mice on the same diet (Figure 6A).

The expression of *Srebf1 and Srebf2*, as well as *Dgat2*, *Soat2* and *Plin2* were downregulated in *Ces1d^-/-^* mice fed HFD *(*Figure 6B). Similarly, mRNA expression of *Ppara, Pparg2* and their target genes *Acsl1* and *Cidec* were reduced in *Ces1d^-/-^* mice fed HFD (Figure 6B). Protein abundance of PLIN2 was reduced in *Ces1d^-/-^* mice, but ACSL1 protein abundance was not significantly altered (Figure 6C).

*Ces1d^-/-^* mice fed HFD also presented with significantly decreased hepatic expression of *Nr1h3*, *Nr1h2 and Rxra* (Figure 6D), with corresponding downregulation of expression of LXR target genes *Abca1, Cyp7a1* and retinoic acid receptor-related orphan receptor α (RORα) target gene *Cyp39a1* (Figure 6E). There was no difference in *Cyp27a1* mRNA or protein expression between *Ces1d^-/-^* and WT mice fed HFD (Figure 6F). We assessed whether hepatic concentrations of oxysterols that could potentially serve as LXR ligands are affected in livers of *Ces1d^-/-^* mice but did not observe significant differences in total, free or esterified hydroxycholesterols (Figure 6G).

### 3.6. Inhibition of CYP27A1 Alleviates Ces1d-Induced TG Accumulation in Mouse Primary Hepatocytes

**Figure 7:**
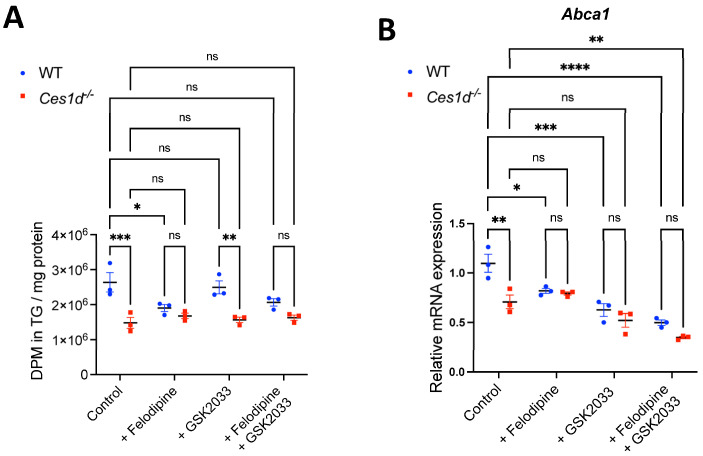
Inhibition of CYP27A1 decreases oleate induced TG accumulation in WT but not Ces1d-deficient mouse primary hepatocytes. Hepatocytes prepared from WT and Ces1d-deficient mice were incubated with 0.4 mM [^3^H]OA complexed with 0.5% BSA for 12 h ± CYP27A1 inhibitor Felodipine, LXR antagonist GSK2033 or both Felodipine and GSK2023 together and incorporation of OA into TG was determined following lipid extraction and TLC using Bioscan radio-TLC Imaging Scanner. Hepatocytes incubated with non-labeled OA ± Felodipine, GSK2023 or both Felodipine and GSK2023 were used for mRNA isolation and determination of *Abca1* (LXR target gene) expression. Results are presented as the ratio of expression of a given gene to cyclophilin. Data are means ± SEM of three independent experiments performed in triplicate. ns, not significant, * *p* < 0.05, ** *p* < 0.01, *** *p* < 0.001 and **** *p* < 0.0001. (**A**) Esterification of [^3^H]OA into TG in the presence and absence of CYP27A1 and LXR inhibitors. (**B**) Relative mRNA expression of *Abca1*.

Because Ces1d activity appears to upregulate LXR expression and neutral lipid accretion, we hypothesized that inhibition of CYP27A1, which produces a ligand for LXR, or antagonism of LXR would attenuate lipid accretion in WT but not in Ces1d-deficient hepatocytes. Indeed, treatment with the CYP27A1 inhibitor Felodipine reduced OA incorporation into TG in WT primary hepatocytes but did not affect OA incorporation into TG in Ces1d-deficient hepatocytes (Figure 7A). LXR antagonist did not reduce TG synthesis in either WT or Ces1d-deficient hepatocytes (Figure 7A) but decreased expression of LXR target gene *Abca1* (Figure 7B).

## 4. Discussion

MASLD is a common obesity-associated liver pathology. The hallmark of MASLD is the accumulation of neutral lipids, TG in particular. Elevated CES1 expression was observed in patients with steatosis and MASH [9], and inactivation of CES1 or its mouse ortholog Ces1d prevents hepatic steatosis [10,13]. Direct role of CES1 in lipid accretion was demonstrated by expression of CES1 in various cell lines where increased CES1 activity resulted in increased TG content accompanied by the presence of larger LDs [12]. Intriguingly, CES1/Ces1d possesses a neutral lipid hydrolase activity [11,30], and therefore, it is unclear how lipid hydrolysis by this enzyme could contribute to lipid accretion, but a possibility exists that CES1/Ces1 provides substrates for specific esterification pathways and/or ligands for nuclear receptors that regulate these pathways. Fatty acids used for hepatic TG formation can be synthesized by *de novo* lipogenesis, or obtained through turnover of intracellular lipids, or can be of extracellular origin such as fatty acids released by lipolysis of TG stored in adipose tissue or fatty acids obtained by catabolism of lipids from endocytosed lipoproteins [5]. In the current study, we have interrogated the mechanism of CES1 and Ces1d in TG/LD homeostasis in the presence of exogenous fatty acid supply. Our data show that incubation of CES1 cells with OA resulted in increased TG synthesis and storage in larger LDs compared to cells lacking CES1, thus suggesting a role of CES1 in the fatty acid esterification pathway. A possible mechanism that may explain the increased exogenous fatty acid esterification into TG in CES1 cells is the upregulation of expression of the Kennedy pathway genes *Agpat4*, *Lpin1*, *Dgat1* and *Dgat2*. DGAT1 and DGAT2 catalyze acyl-CoA-dependent TG synthesis in mammalian cells. DGAT1 activity synthesizes TG that is stored in numerous small LDs, while DGAT2 activity is involved in LD expansion leading to larger LDs [31,32]. Both DGATs have been reported to esterify fatty acids derived from both endogenous and exogenous sources to form TG in hepatocytes [33]. The ablation of mouse *Ces1d* gene expression yields small LDs in hepatocytes, while expression of CES1 results in larger LDs [13]. This led us to test the hypothesis that CES1 provides substrates for DGAT2-mediated TG synthesis and LD expansion. While in control cells DGAT2 could compensate for TG synthesis when DGAT1 was inhibited, this was not observed in CES1 cells where inhibition of either DGAT resulted in lower TG synthesis. Inhibition of DGAT2 significantly increased the production of small-sized LDs in CES1 cells compared to vehicle treated CES1 cells. Therefore, CES1 activity appears to augment a pathway that preferentially supports DGAT2 catalyzed TG synthesis. This is in agreement with studies suggesting that DGAT2 provides TG for VLDL assembly [34,35], and similarly, CES1/Ces1d supports VLDL assembly, while CES1/Ces1d inactivation decreases VLDL assembly and lowers plasma TG concentration [10,11,13].

SREBP1c is a key regulator of de novo lipogenesis [36], and its mRNA expression was increased in CES1 cells as was its target gene *Scd1* but expression of other SREBP1c-target genes *Acaca* and *Fasn* or the abundance of the encoded proteins (ACC1 and FASN) were not altered. Transcriptional regulation of genes involved in hepatic lipogenesis is under the control of both LXRα/β and SREBP1c [37]. An LXR response element has been identified in the *Srebp1c* promoter and an LXR synthetic agonist induced *Srebf1c* expression, lipogenesis, steatosis and VLDL secretion [38]. LXRα/β play a central role in promoting fatty acid esterification into TG [39]. We examined whether LXR activation is responsible for the augmented TG synthesis in CES1 cells. We observed upregulation in the expression of *Nr1h3* and *Nr1h2* and their shared target gene *Abca1* in CES1 cells. Inactivation of LXR reduced fatty acid esterification in CES1 cells to control cell levels, suggesting that CES1 activity may indeed contribute to LXR activation. Activation of LXR requires oxysterols, including 27-hydroxycholesterol (27-HC) [25,40,41]. CYP27A1 (sterol 27-hydroxylase) catalyzes various sequential steps of the oxysterol and bile acid synthesis pathways and is responsible for 27-HC synthesis [28]. We observed upregulation of *Cyp27a1* mRNA expression in CES1 cells; however, CYP27A1 protein abundance was similar to control cells. Recent studies found that *CES1* knockdown in THP1 macrophages results in decreased expression of CYP27A1- and LXRα-regulated processes due to decreased CYP27A1-mediated endogenous oxysterol production [25]. However, in our study, hepatic CYP27A1 expression and its ligand production were found to be unaltered in Ces1d-deficient hepatocytes, suggesting differential regulation in various cell types. On the other hand, mRNA expression of genes encoding SREBP1c, LXRα/β, RXR, PPARα and PPARγ were found to be markedly reduced in Ces1d-deficient livers. Together, these findings suggest that *Ces1d* ablation affects nuclear receptor transcriptional regulatory function, possibly by limiting the receptor abundance. Our study also showed that the expression of PPARγ target genes was altered in both CES1/Ces1d-expressing and knockout models. In CES1 cells, expression of PPARγ target genes, i.e., Plin2,* Acsl1* and *Cidec* were upregulated and several of the same genes are downregulated in Ces1d-deficient livers. Cide proteins are playing a key role in LD growth and Cidec has been shown to promote hepatic steatosis [42]. Increased *Cidec* expression in CES1 cells and decreased *Cidec* expression in Ces1d ko livers provides additional mechanistic explanation for the observed LD morphologies. Upregulation of PPARγ pathway agrees with studies in which expression of active CES1 but not inactive CES1 in livers lacking endogenous Ces1d reduced fatty acid oxidation and increased TG accumulation [13]. Mice lacking Ces1d showed an increase in hepatic fatty acid oxidation and improved hepatic insulin sensitivity [10,11]. Intriguingly, ACSL1 directs acyl chains to mitochondrial β-oxidation [43] and it is not clear why the expression of the gene encoding this enzyme is increased in cells with augmented TG synthesis (CES1 cells) and decreased in cells with increased fatty acid oxidation (Ces1d-deficient hepatocytes). While ACSL1 expression (both mRNA and protein) is increased in CES1 cells, decreased *Acsl1* expression in Ces1d-deficient livers did not translate to decreased ACSL1 protein, suggesting a more complex regulation of hepatic ACSL1 in fasted mice.

## 5. Conclusions

**Figure 8:**
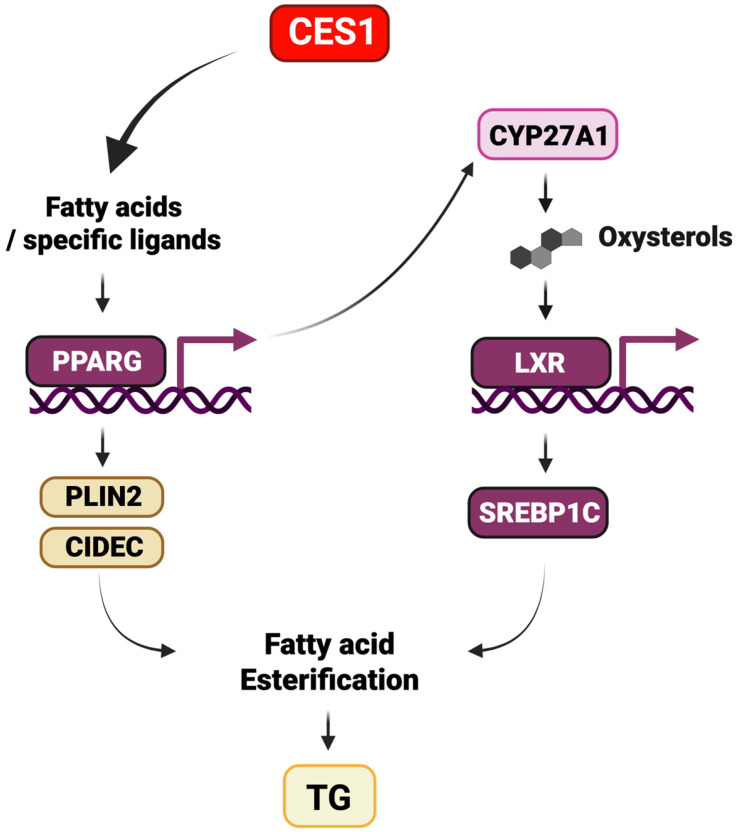
Model of CES1 mediated fatty acid esterification. Lipolytic action of CES1 releases fatty acids which serve as source of ligands for PPARγ regulated processes including activation of lipid storage and LD growth gene expression. Additionally, CES1 activity modulates LXR-SREBP1c pathway to promote TG synthesis potentially through the activation of PPARγ.

In conclusion, in this study, we demonstrated that CES1/Ces1d mediates hepatic TG/LD accumulation through activation of the fatty acid esterification pathway (Figure 8). Although our data suggest the importance of the LXR-SREBP1c pathway in mediating fatty acid esterification by CES1/Ces1d, CES1/Ces1d also appears to regulate PPARγ transcriptional activation of lipid metabolism. The results from this study further confirm that the development of specific CES1 inhibitors could be beneficial in lowering hepatic fat accumulation.

## Data Availability

No additional data in addition to those presented in this manuscript and supplementary data were generated.

## Supplementary Materials

The following supporting information can be downloaded at https://www.mdpi.com/article/10.3390/cells14191548/s1, Figure S1: Expression of CES1 protein in human liver (HL) and McArdle RH7777 cells stably transfected with an empty pNeo vector or a pNeo vector containing CES1 cDNA; Figure S2: Incorporation of OA into cholesteryl esters and phospholipids and storage of lipids in LDs during 12 incubation; Figure S3: Immunoblot analysis of fatty acid synthetic enzymes in pNeo and CES1 cells after 12 h OA incubation; Figure S4: Incorporation of OA into phospholipids in cells treated with PPARγ antagonist or LXR antagonist and CYP27A1 inhibitor; Table S1: Primers used for quantitative PCR analysis.

## Abbreviations

The following abbreviations are used in this manuscript:

ACSL	Acyl-CoA synthetase
BSA	Bovine serum albumin
CES/Ces	Carboxyl esterase
DGAT	Diacylglycerol acyltransferase
FA	Fatty acid
HFD	High fat diet
LDs	Lipid droplets
MASLD	Metabolic dysfunction-associated steatotic liver disease
MASH	Metabolic dysfunction-associated steatohepatitis
OA	Oleic acid
TG	Triacylglycerol
TLC	Thin-layer chromatography
VLDL	Very low-density lipoproteins
