# Supplementary material for: CES1 Increases Hepatic Triacylglycerol Synthesis Through Activation of PPARγ, LXR and SREBP1c
Source: Cells. 2025 Oct 3;14(19):1548. doi: 10.3390/cells14191548 (PMC12524082; doi:10.3390/cells14191548)
Supplement: Supplementary file 1 [file cells-14-01548-s001.zip › cells-3840707-supplementary.pdf]

Supplemental Table S1. Primers used for quantitative PCR analysis

Gene	Rat specific primer sequence
Abca1	F: 5'- GCCTGTTGGTGATCATCTTG - 3'
	R: 5'- ACCACGCTGGGGTCACTAT - 3'
Acaca	F: 5' - GATGATCAAGGCCAGCTTGT - 3'
	R: 5' - CAGGCTACCATGCCAATCTC - 3'
Acsl1	F: 5' - GGTTACACACGGGGGACAT - 3'
	R: 5' - TCCTGTCGATAATCTTCAAGGTG - 3'
Agpat4	F: 5' - AGTAGAGGCAGTTTCTGAACCCTA -3'
	R: 5'- AGAAAGATCCGGGGCTCA - 3'
Cpt1a	F: 5'- ACAATGGGACATTCCAGGAG - 3'
	R: 5' - AAAGACTGGCGCTGCTCA - 3'
Ppia	F: 5' - TCCAAAGACAGCAGAAAACTTTCG - 3'
	R: 5' - TCTTCTTGCTGGTCTTGCCATTCC - 3'
Cyp27a1	F: 5' - TTCCAGCTATTTCTACGAGGCTAT - 3'
	R: 5' - CCGTACTTGGCCTTGTTCA - 3'
Cyp39a1	F: 5' - GCTTCTCTGGCCAATGCT - 3'
	R: 5' - CTGTGCAGATCGGGATGAG - 3'
Dgat1	F: 5' - TACGGCGGGTTCTTGAGAT - 3'
	R: 5' - CGTGAATAGTCCATGTCCTTGA - 3'
Dgat2	F: 5' - GCTGGTGCCCTACTCCAAG - 3'
	R: 5' - AGCTTAGGGACGGTGATGG - 3'
Fasn	F: 5'- GTGGACATGGTCACAGAGA - 3'
	R: 5' - CGCTTAGGCAACCCATAGAG - 3'
Lpin1	F: 5' - AGTCTTGCCACTTCCATGCT - 3'
	R: 5' - GCTCAGAATCACTTTTTGGTGTT - 3'
Plin2	F: 5' - TTTGCTCGTCTCTCAGCTCTC - 3'
	R: 5' - CCACTGCTACTGATGCCATTT - 3'
Ppara	F: 5' - TGCGGACTACCAGTACTTAGGG - 3'
	R: 5' - GGAAGCTGGAGAGAGGGTGT - 3'
Pparg	F: 5' - CCCAATGGTTGCTGATTACA - 3'
	R: 5' - GGACGCAGGCTCTACTTTGA - 3'
Fabp1	F: 5' - CTTCTCCGGCAAGTACCAAG - 3'
	R: 5' - TTCCCTTTCTGGATGAGGTC - 3'
Cidec	F: 5’ - GAGGACCTCCTGGGCAAG - 3’
	R: 5’ - ATTGTGCCATCTTCCTCCAA - 3’
Scd1	F: 5' - CATGTCTGACCTGAAAGCTGA -3'
	R: 5' - CAGGAGGCCAGGCTTGTAG - 3'
Soat2	F: 5' - CCCAGACCTGGTACAATGGA - 3'
	R: 5' - CTGTGCTTGCTCCAGACACT - 3'

Supplemental Figures

Figure S1

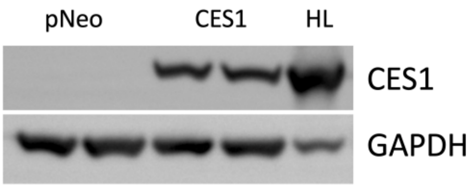

Expression of CES1 protein in human liver (HL) and McArdle RH7777 cells stably transfected with an empty pNeo vector or a pNeo vector containing CES1 cDNA.
GAPDH was used as a loading control.

Figure S2

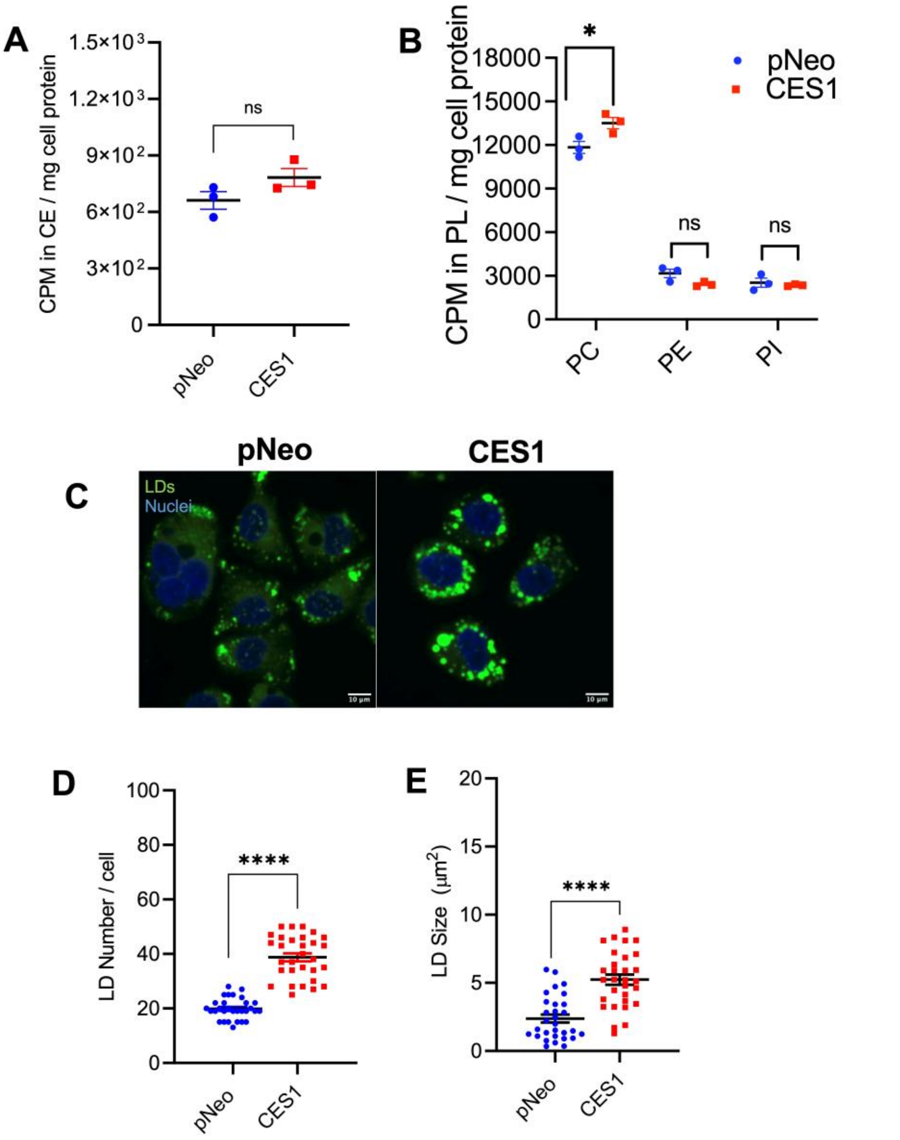

Incorporation of 0.4 mM [^{3}H]OA into cholesteryl esters (CE) (**A**) and phospholipids (**B**) in pNeo and CES1 cells during 12 h incubation. PC, phosphatidylcholine; PE, phosphatidylethanolamine; PI, phosphatidylinositol. Lipid droplet (LD) formation (**C**), number (**D**) and size (**E**) in pNeo and CES1 cells after 12 h incubation with 0.4 mM OA.

Figure S3

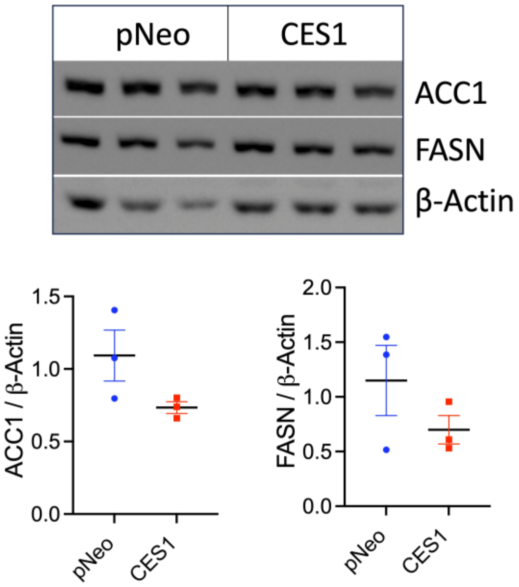

Immunoblot analysis of fatty acid synthetic enzymes in pNeo and CES1 cells after 12 h OA incubation. β-Actin was used as a loading control

Figure S4

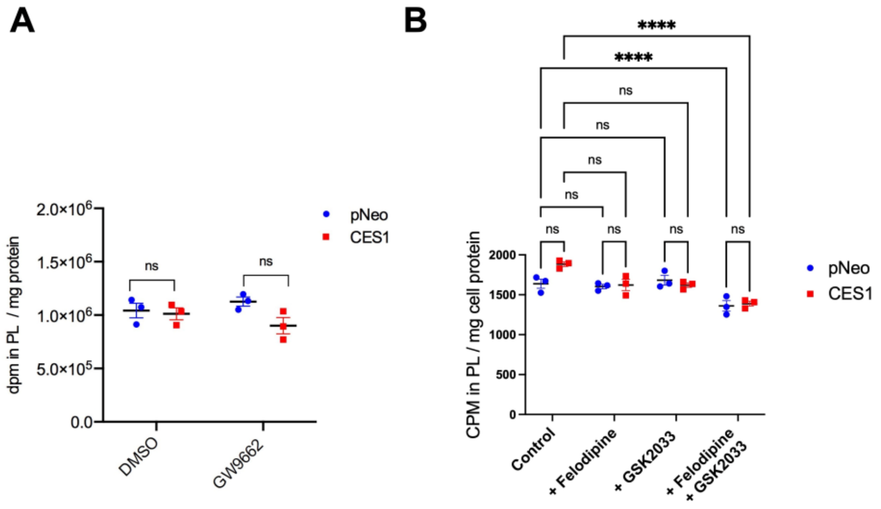

Incorporation of OA into phospholipids in cells treated with PPARγ antagonist (**A**) or LXR antagonist and CYP27A1 inhibitor (**B**).
